# Effects of 2′-O-Methyl Nucleotide Substitution on *Eco*RI Endonuclease Cleavage Activities

**DOI:** 10.1371/journal.pone.0077111

**Published:** 2013-10-23

**Authors:** Guojie Zhao, Bin Zhao, Zhaoxue Tong, Runqing Mu, Yifu Guan

**Affiliations:** 1 Key Laboratory of Medical Cell Biology (Ministry of Education), Department of Biochemistry and Molecular Biology, China Medical University, Shenyang, China; 2 Department of Movement Science, Shenyang Sport University, Shenyang, China; 3 Department of Laboratory Medicine, First Affiliated Hospital, China Medical University, Shenyang, China; University of Helsinki, Finland

## Abstract

To investigate the effect of sugar pucker conformation on DNA-protein interactions, we used 2′-O-methyl nucleotide (2′-OMeN) to modify the *Eco*RI recognition sequence -TGAATTCT-, and monitored the enzymatic cleavage process using FRET method. The 2′-O-methyl nucleotide has a C3′-endo sugar pucker conformation different from the C2′-endo sugar pucker conformation of native DNA nucleotides. The initial reaction velocities were measured and the kinetic parameters, K_m_ and V_max_ were derived using Michaelis-Menten equation. Experimental results showed that 2′-OMeN substitutions for the *Eco*RI recognition sequence decreased the cleavage efficiency for A2, A3 and T4 substitutions significantly, and 2′-OMeN substitution for T5 residue inhibited the enzymatic activity completely. In contrast, substitutions for G1 and C6 could maintain the original activity. 2′-fluoro nucleic acid (2′-FNA) and locked nucleic acid (LNA) having similar C3′-endo sugar pucker conformation also demonstrated similar enzymatic results. This position-dependent enzymatic cleavage property might be attributed to the phosphate backbone distortion caused by the switch from C2′-endo to C3′-endo sugar pucker conformation, and was interpreted on the basis of the DNA-*Eco*RI structure. These 2′-modified nucleotides could behave as a regulatory element to modulate the enzymatic activity *in vitro*, and this property will have potential applications in genetic engineering and biomedicine.

## Introduction

**Figure 1:**
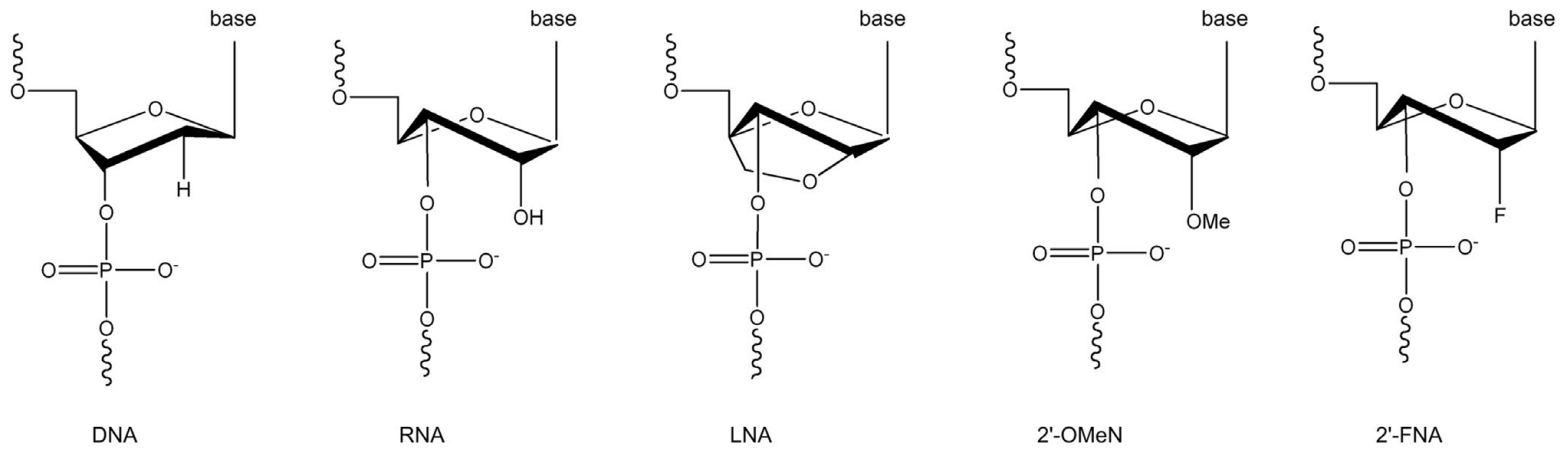
Chemical structures of DNA, RNA, LNA, 2′-OMeN and 2′-FNA.

Nucleic acid chemistry has seen a remarkable progress in developing novel nucleotide derivatives [1]. With chemical modifications on bases, ribose or phosphate group, these nucleotide derivatives have demonstrated many unusual properties. Particularly, the 2′-modified nucleotides have attracted a great attention. Typical examples include 2′-O-methyl nucleotide (2′-OMeN) and locked nucleic acid (LNA). In the case of 2′-OMeN, the –OH group on the C2′ atom of the native nucleotide is replaced by a –OCH_3_ group, generating a larger steric hindrance [2]. LNA is characterized by a 2′-O, 4′-C-methylene-β-D-ribofuranosyl unit which creates a bicyclic ribosyl structure [3]. The most distinctive feature of 2′-OMeN and LNA is that they have a constrained C3′-endo pucker conformation that is dominant in A-form DNA and RNA (Fig. 1). When incorporated into oligonucleotides, LNA demonstrates an enhanced binding affinity toward their complementary DNA targets, whereas 2′-OMeN binds to complementary RNA nucleotides more favorably [4], [5], [6]. Thus, LNA-modified oligonucleotides can improve the identification of mismatched base pairs [7] and resistance against the exonuclease digestion which benefits their lifetimes *in vivo* applications [8], [9]. On the other hand, 2′-OMeN exhibits a faster hybridization dynamics than usual nucleotides [10]. Furthermore, both LNA and 2′-OMeN demonstrate the capability of modulating structure transitions of the G-quadruplexes between the parallel and the anti-parallel folding topologies [11].

These experimental results promote our interest in knowing how these nucleotide derivatives affect the DNA-protein interactions, particularly, interactions with type II restriction endonucleases. Restriction endonuclease *Eco*RI presents a good model suitable for this study, since it has been characterized very well functionally and structurally [12], [13], [14], [15]. *Eco*RI in a homodimeric form recognizes the DNA palindrome sequence -GAATTC- and cleaves the -O3′-P- bond between G1 and A2 positions, generating two DNA fragments with 4-nt tails at the 5′-ends. This recognition is so specific that non-cognate sequences differing from the canonical recognition sequence by as little as one base-pair could be discriminated unambiguously.

**Figure 2:**
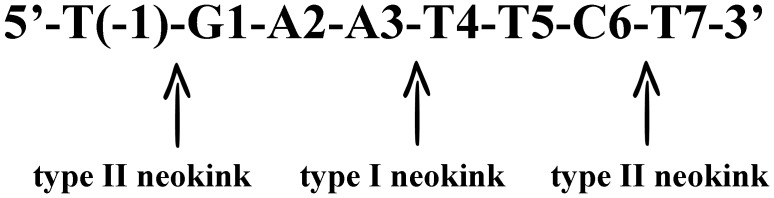
Illustration of three neokinks in *Eco*RI-bonded duplex.

The crystal structure of *Eco*RI-d(TCGCGAATTCGCG) complex has furnished a great amount of information regarding the DNA-*Eco*RI interactions [16]. This global structure shows that, upon binding, the DNA conformation is distorted significantly from the classical B-form. The distortion, however, is not uniformly distributed over the recognition sequence; instead they concentrate in three locations known as neokinks (Fig. 2). Type I neokink lies in the center of the *Eco*RI recognition sequence GAA-TTC, and unwinds systematically the entire duplex by 25°. Thus, type I neokink enlarges the major groove and facilitates the enzyme to access to the substrate. Consequently, type I neokink is truly critical for the enzyme recognition since the α-helixes of *Eco*RI enzyme can only fit into the expanded major groove around type I neokink [16], [17]. Two type II neokinks flank the recognition sequence -GAATTC-. Type II neokinks introduce a large bend of 23° between helical axes. This bend includes a rolling motion toward the minor groove and, importantly, it presents an A–B junction. Considering these structural features, we speculated that site-specific substitutions of 2′-modified nucleotides for the *Eco*RI recognition sequence will change the contacts between the DNA substrates and enzyme *Eco*RI, and consequently, the enzyme-catalyzed cleavage might be enhanced or decreased without scarifying the recognition specificity. Furthermore, recombination DNA technology has become a routine approach to clone DNA fragments and to generate novel DNA constructs. Thus, regulation of enzymatic activity, either the cleavage efficiency or the linkage efficiency, at the designated nucleotide sequences could provide more flexible options for genetic engineering and pharmaceutical applications.

In the present study, we have demonstrated that the enzymatic cleavage activity can be modulated by 2′-OMeN substitutions for the *Eco*RI recognition sequence. Six nucleotides of the *Eco*RI recognition sequence and two nucleotides flanking the recognition sequence have been substituted by 2′-OMeN once a time. The initial reaction velocities were measured and the steady state kinetic parameters, K_m_ and V_max_, were determined. These 2′-OMeN substitutions at different positions showed significantly different effects on the binding affinity and the enzymatic cleavage efficiency. The effects of 2′-OMeN substitutions have been compared with that of LNA, 2′-FNA and other nucleobase derivatives. Mechanisms have been proposed to interpret the unusual enzymatic behavior on the basis of the crystal structure of *Eco*RI-DNA complex. These results suggested that 2′-OMeN substitution could be used as a regulatory element to regulate the *Eco*RI enzymatic activity *in vitro*. This novel property provides an application potential in bioengineering and biomedicine.

## Materials and Methods

### Preparation of Oligonucleotides

In this experiment, three types of oligonucleotides have been used to form the substrate for *Eco*RI enzyme. A 45-mer containing the *Eco*RI recognition sequence -GAATTC- was referred to as template, a 26-mer with a fluorophore (FITC) at the 5′-end was called as F-ON, and a 17-mer with a quencher (Dabcyl) at the 3′-end was called as Q-ON. To evaluate the enzymatic cleavage efficiency, 2′-OMeN, 2′-FNA and LNA were used to modify the *Eco*RI recognition sequence (Table 1). These oligonucleotides were purchased from Sangon Biotech Co. Ltd. (Shanghai, China), and prepared at the concentration of 100 µM in ddH_2_O. These stock solutions were stored in −20°C. In designing the oligonucleotides, we have carefully examined the sequences by using sequence analysis software to eliminate the possible formation of mismatches and hairpins.

**Table 1:** Sequences of oligonucleotides used in this study.

Name	Sequences*
Template	5′-ATACGCATACCTGTGAATTCTGGCTAAAAGCACACGCACGGAGAC
T-T(−1)	5′-ATACGCATACCTGtGAATTCTGGCTAAAAGCACACGCACGGAGAC
T-G1	5′-ATACGCATACCTGTgAATTCTGGCTAAAAGCACACGCACGGAGAC
T-A2	5′-ATACGCATACCTGTGaATTCTGGCTAAAAGCACACGCACGGAGAC
T-A3	5′-ATACGCATACCTGTGAaTTCTGGCTAAAAGCACACGCACGGAGAC
T-T4	5′-ATACGCATACCTGTGAAtTCTGGCTAAAAGCACACGCACGGAGAC
T-T5	5′-ATACGCATACCTGTGAATtCTGGCTAAAAGCACACGCACGGAGAC
T-C6	5′-ATACGCATACCTGTGAATTcTGGCTAAAAGCACACGCACGGAGAC
T-T7	5′-ATACGCATACCTGTGAATTCtGGCTAAAAGCACACGCACGGAGAC
F-ON	3′-TATGCGTATGGACACTTAAGACCGAT-FITC
Q-ON	3′-Dabcyl-TCGTGTGCGTGCCTCTG

* The nucleotide derivatives 2′-OMeN, 2′-FNA and LNA are in lower case, and DNA nucleotides are in upper case, respectively. F-ON is 5′ labeled by FITC. Q-ON is 3′ labeled by Dabcyl. For convenience, the sequences of F-ON and Q-ON are written from the 3′-end to the 5′-end. The *Eco*RI recognition sequence is underlined.

### FRET Analysis of Enzymatic Cleavage

**Figure 3:**
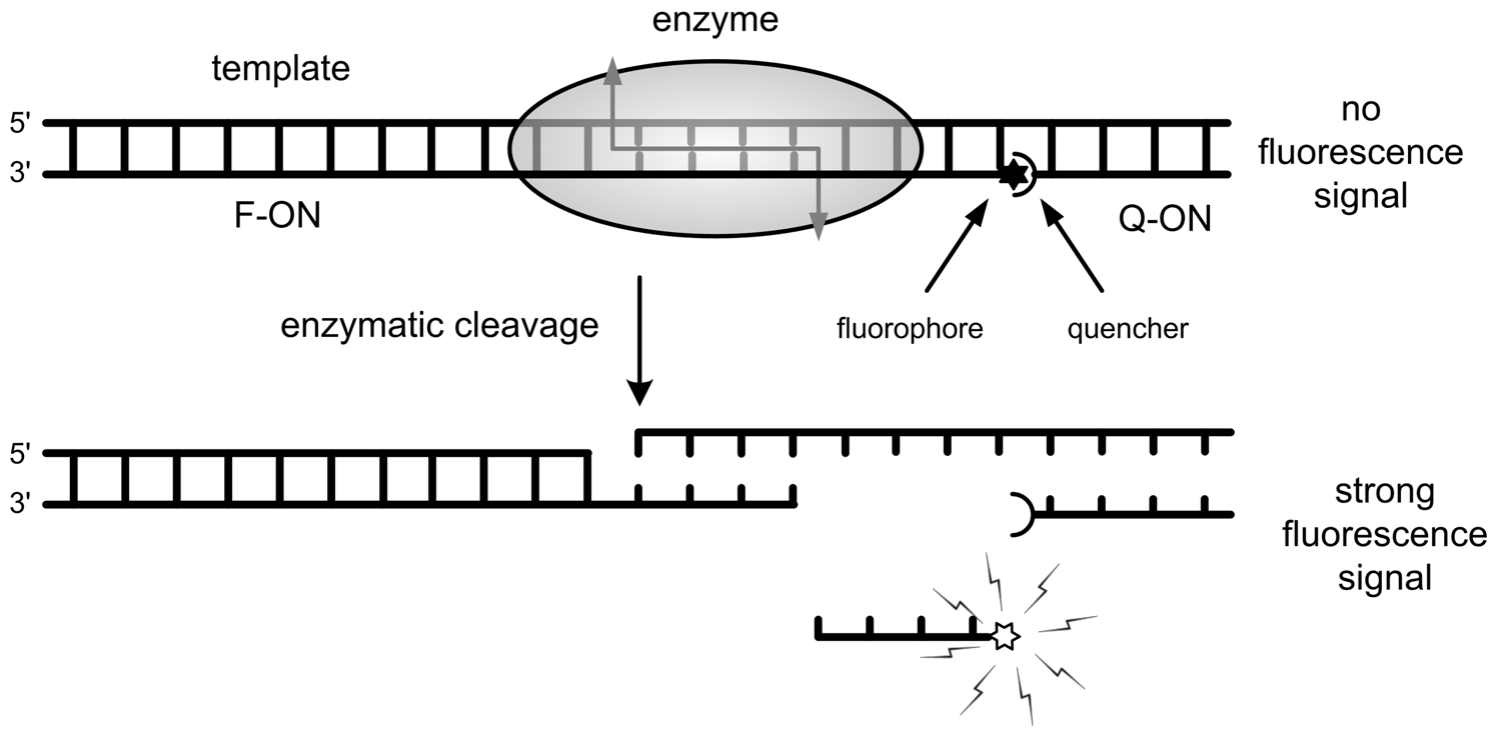
Illustrative diagram showing the principle of the FRET-based assay for endonuclease cleavage measurement.

The substrate of enzyme *Eco*RI is composed of the template, F-ON, and Q-ON at the same concentration of 50 nM in 100 µl buffer containing 50 mM Tris, 10 mM MgCl_2_, 1 mM DTT, and 100 mM NaCl (pH 7.5). The template and F-ON were mixed first, and fluorescence was measured as F0. After adding Q-ON, stable *Eco*RI substrates were formed. In this complex, the quencher and the fluorophore were so close in space that there was very low fluorescence signal defined as F1. Once *Eco*RI was added into the substrate solution, it started to cleave the recognition sequence formed by F-ON and the template. The cleaved 5′-portion of F-ON was too short to hybridize firmly with the template and then released, generating a fluorescence signal Ft (Fig. 3). The relative fluorescence intensity (RF) was calculated using formula RF = (Ft−F1)/(F0−F1).

In this experiment, we have performed a heating-annealing cycle to ensure the formation of three-oligonucleotide substrates before measurement. Fluorescence signals were recorded at 37°C on Microplate Reader (Infinite M200, Tecan, USA) with excitation wavelength at 480 nm and emission wavelength at 524 nm. The signals were collected every 15 seconds with an integration time of 20 microseconds, and measurements were performed by monitoring the fluorescence emission with time over ∼20 min.

### Determination of Kinetic Parameters

The substrate was prepared at the concentration of 0.2∼5.0 folds of K_m_, which was estimated from the initial rates of the different substrate concentrations. Initial velocities (V_0_) of each reaction were determined from the slope of the intensity curves of the fluorescence signal with respect to the time. The substrate concentration of 50∼800 nM and the initial velocity were plotted by using non-linear curve fitting and linear (Lineweaver-Burk) curve fitting, and K_m_ and V_max_ were calculated from Michaelis-Menten equation. Data presented here are averaged results of three independent measurements.

## Results

### Concentration Dependence of Enzymatic Cleavage

**Figure 4:**
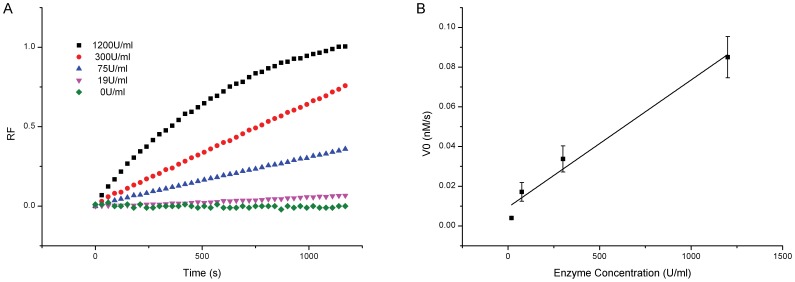
Effects of enzyme concentration on *Eco*RI cleavage. (A) Fluorescence intensity changes with respect to the reaction time for different *Eco*RI enzyme concentrations. (B) The effect of *Eco*RI concentration on the initial reaction velocity for the unsubstituted *Eco*RI recognition sequence.

FRET approach has been established previously for monitoring the restriction endonucease cleavage activity [18], [19], [20], [21]. To validate this method and to determine the enzyme concentration adequate for experiments in the current study, we tested the effects of the enzyme concentration on the cleavage efficiency for the native nucleotide sequence. *Eco*RI concentrations in the range of 19 U/ml to 1200 U/ml were examined. At lower concentrations, the fluorescence intensities increased monotonically as the reaction was progressing, and at higher concentrations, the fluorescence intensities increased rapidly and then became saturated, exhibiting the typical rectangular hyperbolic behavior (Fig. 4A). The initial reaction velocities were then calculated, and showed a linear relationship with the enzyme concentrations (Fig. 4B). Furthermore, by plotting the reaction velocities with respect to the substrate concentrations and fitting these data to the hyperbolic curves, kinetic parameters K_m_ and V_max_ were estimated to be 97.6 nM and 0.103 nM/s, respectively. These values were compatible with the data reported previously with small margins [19], [22]. It has been known that the lengths of the duplex substrates as well as the nucleotides flanking the recognition sequence at 5′- or 3′-end could exert direct or indirect effects on the kinetic values, *e.g.* by affecting the stability of the duplex [23], [24] or the rate-limiting step of the product release [25].

### Position-dependent Enzymatic Cleavage

**Figure 5:**
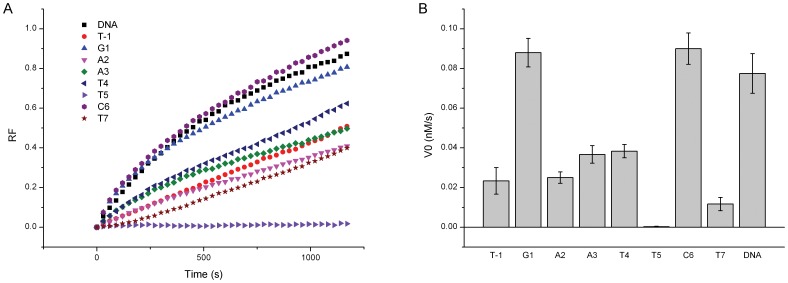
Effects of 2′-OMeN substitution on *Eco*RI cleavage. (A) Fluorescence intensity changes of the 2′-OMeN-modified *Eco*RI sequences with respect to reaction time at the *Eco*RI concentration of 1.2 unit/µl. (B) Initial reaction velocities calculated based on the fluorescence curves in Fig. 5A.

Fig. 5 shows the initial reaction velocities of *Eco*RI when the *Eco*RI recognition sequence nucleotides are modified with 2′-OMeN. In comparison with the unmodified sequence, substitutions at G1 and C6 positions maintaind the initial reaction velocities, whereas substitutions at T(−1), A2, A3, T4 and T7 positions reduced the cleavage efficiency significantly by 50∼80%. The most astonished result was that the *Eco*RI cleavage activity has been completely diminished by 2′-OMeN substitution at T5 position.

**Figure 6:**
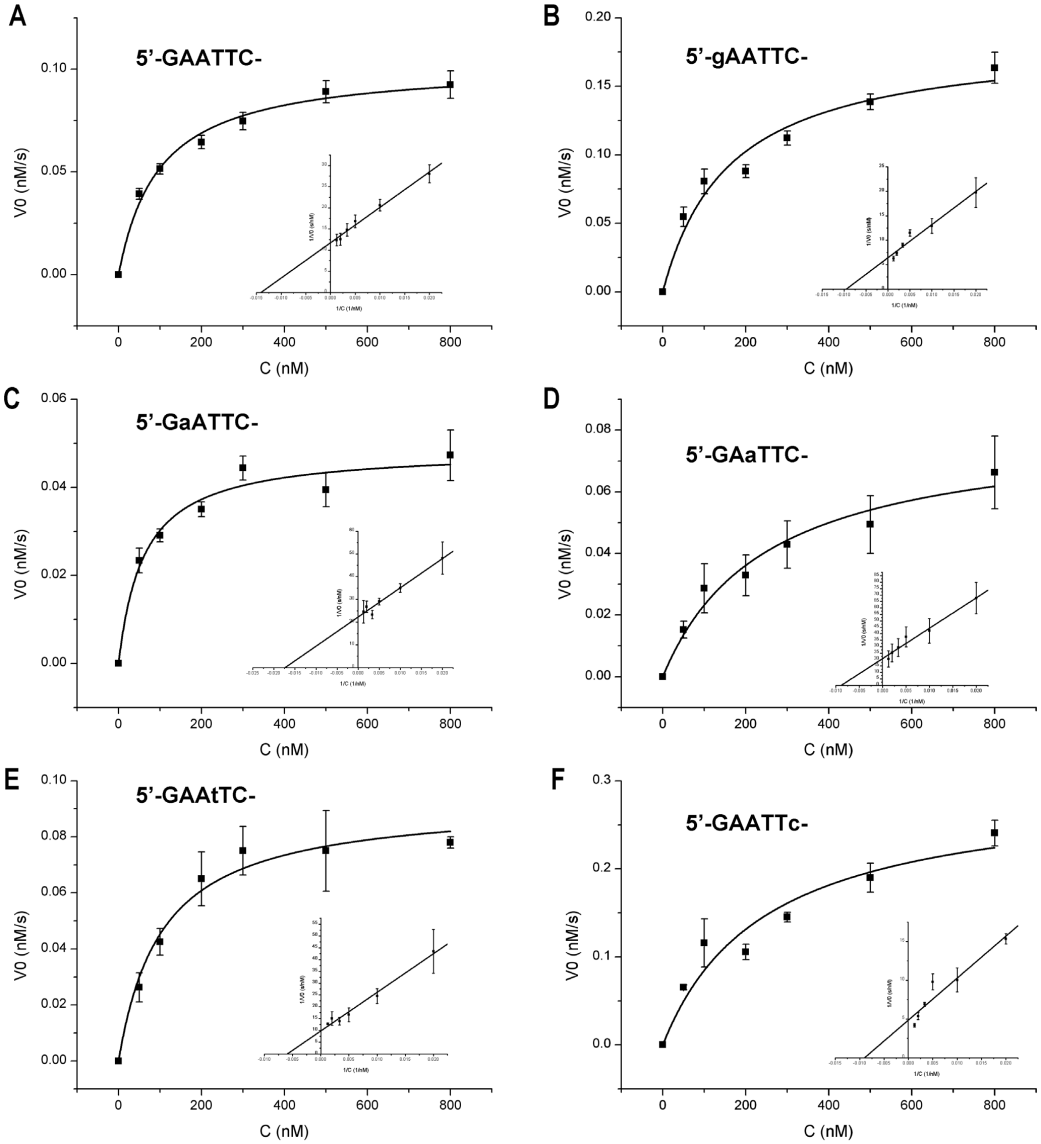
Steady state kinetic analysis of *Eco*RI cleavage of unmodified and 2′-OMeN modified DNA. (A) Unmodified DNA. (B) Modification at G1 position. (C) Modification at A2 position. (D) Modification at A3 position. (E) Modification at T4 position. (F) Modification at C6 position. The line is non-linear curve fitting of the initial velocities at different duplex substrate concentrations. Inset is Lineweaver-Burk plots of the data. Letters and capitals represent 2′-OMeN and DNA nucleotide, respectively.

To understand the molecular interactions involved in this enzymatic cleavage, we have measured the initial reaction velocities of *Eco*RI at different concentrations of the 2′-OMeN modified substrates, and determined their steady state kinetic parameters, K_m_ and V_max_ (Fig. 6 and Table 2). In general, the binding of protein-DNA was reduced in most modified positions, as shown the majority of modified positions presented increased K_m_, though some of increases were small. The V_max_ values increased for G1 and C6 positions, while substitutions at other positions showed decreased activities by different percentages. The ability to discriminate substrate, also referred to as the specificity constant, can be evaluated by V_max_/K_m_. In comparison with the control (the unsubstituted substrate), the values of V_max_/K_m_ varied from the lowest 0.3 of A3 position to the highest 1.2 of C6 position.

**Table 2:** Kinetic parameters for different 2′-OMeN substituted *Eco*RI sequences.

Substitution position	K_m_ (nM)	V_max_ (nM/s)	V_max_/K_m_ (1/s)
GAATTC	97.6	0.103	0.0011 (1.0)
gAATTC	161.8	0.185	0.0011 (1.1)
GaATTC	60.9	0.049	0.0008 (0.8)
GAaTTC	248.4	0.081	0.0003 (0.3)
GAAtTC	104.6	0.093	0.0009 (0.8)
GAATtC	N/A	N/A	N/A
GAATTc	246.2	0.293	0.0012 (1.1)

Letters and capitals represent 2′-OMeN and DNA nucleotide, respectively. In parentheses are the relative values of modified substrate compared to unmodified control.

## Discussion

To identify the interactions involved in determining the sequence-specified recognition, different base derivatives have been utilized to make site-specific substitutions of d(-GAATTC-) sequence, including hypoxanthine [22], 5-Br-C [26], 5-Br-dC [22], 5-Br-U [22], 8-Br-dA [27], 2,6-diaminopurine [22], 6-methyl-dA [22], 5-methyl-dA [22] and 7-deaza-dA [27], [28], [29]. dU or 5-Br-dT substitution has concluded that T5 position is more important than T4 position, and it is possibly attributed to the steric hindrance of 5-CH_3_ group on thymine residue [22]. Modification at either A2 or A3 position by 6-methyl-dA has led to no enzymatic cleavage [22]. Furthermore, phosphate derivatives of methylphosphonate, O-ethyl-phosphotriester and phosphorothioate have been used to modify the phosphate groups of the *Eco*RI recognition sequence. Each of these chiral derivatives creates two different spatial orientations of the substituents at the phosphorous atom and results in enzymatic activity variations from complete inactivation to significant enhancement. The negative charge dispersion and reactivity of different chemical groups have been considered to be responsible for these changes [30], [31], [32], [33].

In comparison with these investigations, the effects of 2′-modified nucleotide derivatives on the recognition and hydrolytic cleavage of *Eco*RI have much less explored. 2′-fluoro-2′-deoxyguanosine (2′-F-dG) and 2′-chloro-2′-deoxyguanosine (2′-Cl-dG) have been the only two 2′-modified nucleotide derivatives so far, and only the G1 residue of the *Eco*RI sequence has been modified. Modification of the residue flanking the recognition sequence at the 3′-end by L-nucleotide (ribose in L-homochirality) has shown different enzymatic behavior from the normal one [19]. In structure biology, six torsion angles along the phosphate backbone -P-O5′-C5′-C4′-C3′-O3′-P- of DNA strand, namely α, β, γ, δ, ε and ζ, are used to characterize the backbone conformations of DNA and RNA, and each one has been restricted in a well defined range for different conformations of DNA duplexes. The C3′ atom is directly involved in torsion angles δ and ε, thus, the C3′-endo sugar pucker conformation of 2′-modified nucleotides will inevitably alter the duplex phosphate backbone, and consequently, create different effects on either the binding affinity with the substrate or the cleavage efficiency of *Eco*RI.

Crystal structures of several *Eco*RI-DNA complexes have been reported. They have provided a structural basis for interpreting the molecular interactions involved in enzymatic cleavage. The *Eco*RI-d(TCGCGAATTCGCG) complex has shown a tight and complementary contact between enzyme and the major groove of the DNA duplex. There are twelve *Eco*RI-purine hydrogen bonds as well as a set of van der Waals contacts responsible for the specific recognition. It has also been found that Lys130 and Arg203 form hydrogen bonds with phosphate groups [34]. Furthermore, each strand of the DNA duplex interacts with two subunits of homodimeric *Eco*RI with comparable energies [35].

### G1 Position

G1 is just on the edge of the type II neokink. Crystal structures of several *Eco*RI-DNA complexes have revealed that DNA duplex is surrounded by both direct and indirect recognitions, *i.e.* both phosphate backbone and bases are bonded by functional elements of enzyme. The O6 and N7 atoms of G1 nucleotide interact with Arg200 of *Eco*RI. Both of the two non-bridging oxygen atoms of G1 phosphate group are bonded with the conserved residues Lys89, Lys148 and Asn149. Previous study has reported that hypoxanthine substitution of G1 increased both K_m_ and k_cat_ [22]. Hypoxanthine substitution removed away the C2-NH_2_ group from minor groove and left functional groups in major groove untouched. It has been explained in terms of the backbone conformation alternation of type II neokink caused by hypoxanthine substitution in minor groove. Our results showed that the 2′-OMeN substitution for G1 nucleotide also increased K_m_ as well as V_max_, suggesting a reduced binding affinity and an enhanced cleavage efficiency. Since the G1 position is just on the edge of type II neokink and must experience the A–B form transition. Thus, 2′-OMeN substitution at G1 position might arrange the substrate backbone to an energetically favored conformation, facilitating the organization of the catalytic center in the catalytic step and benefiting the *Eco*RI cleavage. Accelerated product releasing may also attribute to the increased V_max_.

A previous study by Gohda et al. has selected 2′-fluoro-2′-deoxyguanosine (2′-F-dG), 2′-chloro-2′-deoxyguanosine (2′-Cl-dG) and guanosine (rG) to substitute G1 residue of the d(GGAATTCC) sequence, and examined their effects on *Eco*RI cleavage activities [36]. 2′-F-dG modified sequence has shown a similar property to that of normal DNA substrate: 100% cleavage for both the unmodified and the 2′-F-dG modified d(GG*AATTCC) sequence in 60 min. In contrast, rG and 2′-Cl-dG modifications have repressed the cleavage differently: only 5% for rG and no cleavage at all for 2′-Cl-dG in 24 h. Since these four octamers exhibit similar binding affinity (10^−6^–10^−7^ M), the differences in cleavage activity may be attributed to the catalytic reaction instead of the binding process. They have also used ^31^P-NMR method to analyze the change of the phosphate backbone conformation, and observed that the order of the conformation changes (2′-Cl-dG>rG>2′-F-dG) coincided with the observed cleavage activity as well as the van der Waals volumes of the 2′-substituents (Cl>OH>F). Thus, the steric hindrance of the 2′-substituent has been considered to be the cause of the conformational changes of the phosphate backbone and consequent changes of enzymatic activities. They have also used the 2′-Cl-dG substituted and rG substituted octamers as inhibitors in this study, and concluded that the substituted octamers behaved like a competitive inhibitor [36].

**Figure 7:**
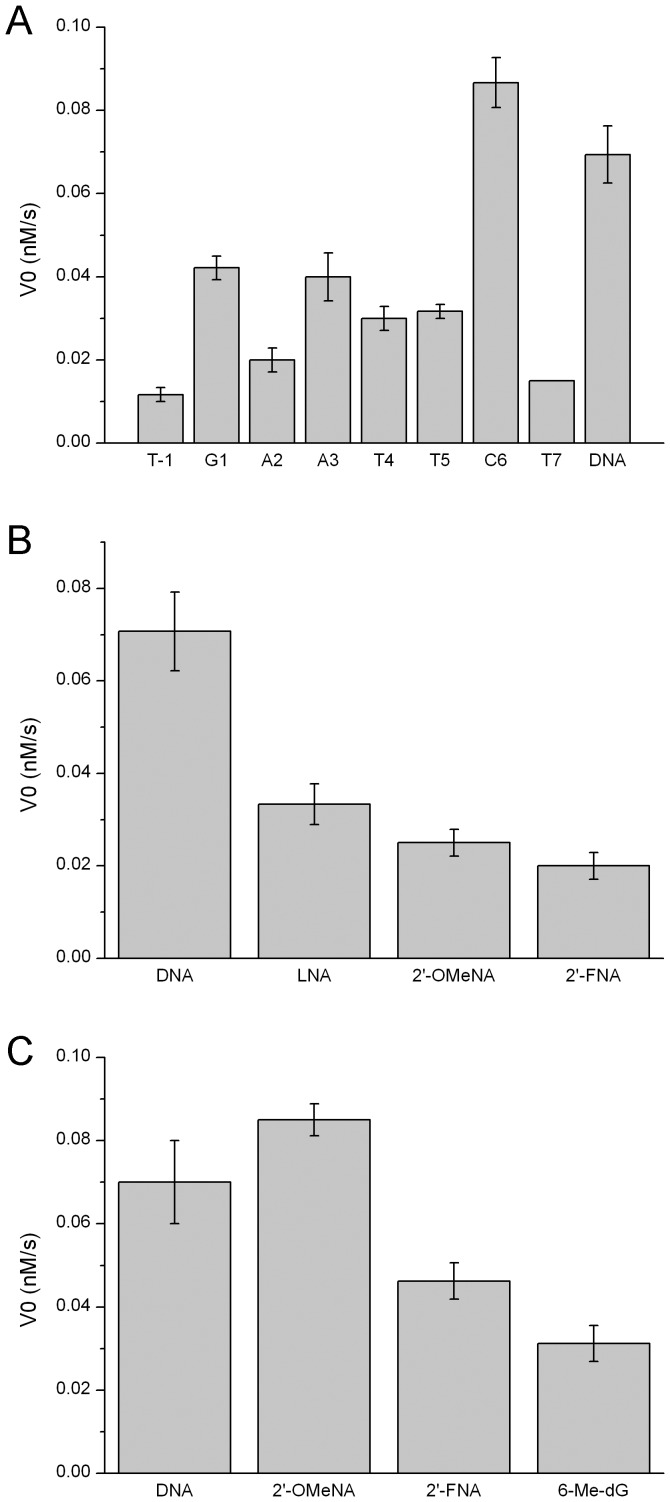
Effects of other analog substitutions on *Eco*RI cleavage. (A) The effects of 2′-FNA substitution at different positions on the reaction initial velocity. (B) Comparison of initial reaction velocities of different nucleotide derivatives at G1 position. (C) Comparison of initial reaction velocities of different nucleotide derivatives at A2 position.

In the currrent study, we also made substitutions at this G1 position using 2′-FNA. This 2′-FNA substitution reduced the initial reaction velocity by about ∼30% (Fig. 7A). Considering the difference in size and charge of substituent group, the electronegativity as well as the van der Waals radius might compromise the favorable conformation by 2′-fluoro modification. We also used the base derivative O6-Me-dG to substitute for G1 nucleotide and observed a 50% reduction in the cleavage efficiency (Fig. 7B). The methylation of O6-dG can disrupt the hydrogen bond between O6 and Arg200. The steric hindrance of methyl group may also cause a damage of adjacent interactions. Therefore, O6-Me-dG substitution destabilized enzyme-DNA complex and decreased cleavage rate. Previous studies have revealed that O6 and N7 of G1 residue interacted with Arg200 of *Eco*RI [17] and removal of N7 by substitution of 7-deaza-dG decreased the cleavage significantly [28]. In comparison with these experimental data, the current data might suggest that sugar pucker conformation switch has less effect in this position on regulating the enzymatic cleavage than that of the nucleobase modifications.

### A2 Position

A2 position plays an important role in sequence recognition and cleavage. In the catalytic center, N6 and N7 of A2 nucleotide interact with Glu144 and Arg145 through hydrogen bonds which contribute to the specific recognition of adenine. The phosphate of A2 is also fixed by Asp91 and Glu111 through Mg^2+^ for hydrolyzing phosphoester bond. 2′-OMeN substitution for A2 residue has reduced both K_m_ and V_max_. The specificity constant decreased slightly due mainly to the decreased V_max_. The phenomena of better binding alone with the reduced V_max_ have also observed in other studies [37], [38]. The alteration of A2 phosphate group in the catalytic center can exert direct inhibitory effect on the cleavage. Structural analysis has pinpointed another possibility. The phosphate group of -A2pA3- forms a hydrogen bond with His114. The His114 distrubance induced by backbone change could interfere with the adjacent residues Glu111 and Lys113 which are the elements at the catalytic center. Moreover, it has been proposed that Gln115, another key residue adjacent to His114, plays an important role in coupling the specific DNA binding to the catalytic cleavage [39]. Though V_max_ of this position presented the lowest value of all six substituted positions except T5, the reduced extent is still below our expectation when taking into account many of the most important residues mentioned above. This gives us an impression that an elaborate and flexible interaction network must have the ability to compensate the reduced V_max_. In addition, we have proposed another interpretation. Based on the crystal structure, the most possible residue to interact with C2′ moiety of A2 position is Arg145, since the distance of C2′ atom to the nearby residue Arg145 of *Eco*RI was determined to be 4.69 Å (1CKQ.pdb). The electronegativity of 2′-OMe can also increase its interaction with this basic amino acid. Since both the bases and backbones of A2 position interact with functional groups of *Eco*RI in a more complex format, the retardation by C2′ derivatives might be a mixed effect. Further studies are under conduct currently.

No literature could be found regarding the substitution of the A2 residue by 2′-modified nucleotide derivatives so far, thus, we used chiral methylphosphonates in previous studies for comparison [30]. Chiral methylphosphonates, namely Sp-Pm and Rp-Pm, have replaced the phosphate group of -A2pA3-. Experimental results have shown that the binding affinities were impaired by both Sp-Pm and Rp-Pm substitutions, whereas the cleavage efficiencies were also reduced to quite different extent. It has been proposed that the Rp-oxygen had no binding partner while Sp-oxygen was hydrogen-bounded with His114 and a localized water molecule [30]. The C3′-endo conformation of the 2′-OMeN substitution at the A2 position in the present study could influence the local backbone of -A2pA3-, thus altered the position of His114. We also made 2′-FNA and LNA substitutions for A2 residue, and observed the reduced cleavage ability by about 50∼60% (Fig. 7C). Because they share the same C3′-endo sugar pucker conformation as 2′-OMeN [4], [40], [41], the backbone distortion may play a major role in this cleavage rate reduction.

### A3 Position

A3 position experiences the central type I neokink which unwinds duplex in -A3pT4- by 25°, resulting in a wider major groove and a narrower minor groove. Since *Eco*RI access DNA duplex through major groove, the enlarged major groove is necessary for enzyme’s entry. In addition, A3 is also the nucleotide whose base can be directly read out by protein, *i.e.* Arg145 forms hydrogen bond with N7 of A3 nucleotide. In previous study, 2,6-diaminopurine (2,6-AP) has been used to substitute for this position and introduced C2-NH_2_ into minor groove, and increased K_m_ and decreased k_cat_ have been observed [22]. It has been suggested that an additional C2-NH_2_ in minor groove as a bulkier obstacle interferes with type I neokink formation. In our study, a significant increase in K_m_ and obvious decrease in V_max_ were observed in the situation of 2′-OMeN substitution at the A3 position, thus gave rise to the lowest specificity constant. C3′-endo sugar pucker facilitate A-form duplexes which have even narrowed major groove in comparison with B-form. This is surely troublesome for the duplex binding by enzyme, and could diminish the binding affinity as well as the cleavage rate. Further crystal structural analysis showed that the distance of the C2′ atom of A3 residue to adjacent residue Ala142 of *Eco*RI is 4.32 Å (1CKQ.pdb). This would also increase a possibility of steric hindrance. In the case of 2′-FNA substitution, the modified substrates showed the reduced cleavage rates by about 40% which is similar to that of 2′-OMeN modification (Fig. 7A). Thus, the retardation by 2′-OMeN and 2′-FNA substitutions might be due mainly to the backbone conformation change which disturb the type I neokink, and inhibit the binding and cleavage of *Eco*RI.

### T4 Position

2′-OMeN substitution for T4 residue increased K_m_ slightly and decreased V_max_ in a small margin, and the specificity constant decreased slightly in comparison with the native substrate. Previous studies have showed that the C5-CH_3_ group of T4 residue interacted with Gly140, Ala142 and Gln115 through hydrophobic interaction [42], and dU substitution for T4 residue diminished the hydrophobic interaction and resulted in an increased K_m_ and almost unchanged k_cat_ which is similar to 2′-OMeN substitution in our study [22]. Thus, 2′-OMeN substitution for T4 residue created a relatively minor impact on the enzymatic cleavage behavior. The interaction of phosphate backbone with Lys117 and Gly116 may be also disturbed by C3′-endo conformation. Similarly, 2′-FNA substitution for T4 residue declined the enzymatic cleavage at a similar level (Fig. 7A).

### T5 Position

To our surprise, 2′-OMeN substitution for T5 residue diminished the *Eco*RI enzymatic activity completely (Fig. 5). No K_m_ and V_max_ could be measured. In previous studies, using dU or 5-Br-dT substitution, T5 residue has been found to be more important than T4 residue which was also true for 2′-OMeN substitution in our study. The dU substitution deleting C5-CH_3_ of T5 but not T4 position has abolished cleavage, which has been suggested to be attributed to the interaction of C5-CH_3_ group, or a conformation alteration of N-glycosidic bonds in dA⋅dU pairing, moreover, in which the paired dA of dU is just involved in the cleavage site. [22]. Both the C5-CH_3_ of T4 and T5 were surrounded by hydrophobic groups of protein functional elements in enzyme-DNA complex crystal structure, such as Gly140, Ala142, and Gln115. However, in our study, the inhibition of cleavage was so strong that hydrophobic interaction is not enough to interpret the observation. It is more likely that this substitution is followed by breakdown of cooperative mechanism, where one alteration causes other more losses of protein-DNA interactions which involved in the elaborated interaction networks of the complex. In this mechanism, the effects can even spread to the unmodified strand. (We will discuss this point later.) Though the T5 position does not fall into the three neokinks of protein-DNA complex, the backbone conformation change by C3′-endo pucker might be an incentive for further breakdown of networks. This explanation has also been suggested by dU substitution at T5 position [22]. In addition, we also pay attention to the C2′ substitution moiety of T5 position. Structural analysis showed that the nearest residue to the C2′ moiety of T5 position is Met137 (3.9 Å (1CKQ.pdb)) which is located at the end of an extended chain, and the –OCH_3_ moiety at C2′ might push extended chain away by steric hindrance of Met137 to disturb key interactions between extended chain and bases. In comparison with 2′-OMeN modification, substitution of the T5 residue by 2′-FNA only decreased but not abolished enzymatic cleavage (Fig. 7A).

### C6 Position

2′-OMeN substitution for C6 residue increased both K_m_ and V_max_ significantly, resulting in an almost unchanged specificity constant. Many base analog substitutions at C6 position have given poor substrates, such as 5-methylcytocine and 5-bromocytocine [22]. It has been suggested that 5-methylation decrease the flexibility and mobility of duplex backbone in C6 position and then reduced catalysis [43], [44]. Since just like G1 residue, C6 residue is on the edge of type II neokink, the flexibility and distortion at this site are very important for the binding and cleavage of *Eco*RI. Thus, the reduced flexibility and mobility of C6 backbone will prevent cleavage. In the case of 2′-OMeN substitution of this study, the C3′-endo conformation of 2′-OMeN could set the local structure to an energetically favored A-form for enzymatic cleavage. Consequently, an almost unchanged cleavage rate was observed in this position. In comparison, 2′-FNA modifications at C6 position produced similar initial reaction velocities to that of 2′-OMeN substitution (Fig. 7A).

### Residues Flanking 5′- and 3′-termini

2′-OMeN substitutions for T(−1) and T7 residues decreased the initial reaction velocities significantly (Fig. 5). These results implied that, in addition to the hexameric sequence, residues flanking the recognition sequence are also important for the hydrolytic cleavage. Crystal structure has suggested that the phosphates of pNpG1 interact with residues in β-bridge, such as Ser86, Ser87 and Lys89. It is believed that the C3′-endo conformation switch at the T(−1) position distorted these local phosphate backbone contacts between *Eco*RI and DNA substrate and changed enzymatic properties. These results are consistent with a previous study that the phosphate contacts at pGAATTC play a crucial role in the recognition [29]. In addition, the effect of unnatural L-nucleotide (ribose in L-homochirality) residues at the 3′-flanking site of the *Eco*RI recognition sequence has been evaluated, showing a larger V_max_ value (2.5 folds) and a smaller K_m_ value (∼3 folds) compared with normal D-nucleotide (ribose in D-homochirality) one. It has been attributed to the L-nucleotide induced preferable conformational alternation for binding and catalysis by *Eco*RI [19]. Moreover, phosphothioate substitution of C6pN has shown that both Sp and Rp substituents give very poor substrates [30]. The interaction of Lys130 has been proposed for the reason. The 2′-OMeN substitution of T7 also presented significantly reduced cleavage rate, and might share similar mechanism with that of phosphothioate substitution.

### Interstranded Interaction

Phosphate derivatives have been extensively studied to manifest the enzymatic cleavage mechanism. R- and S-methylphosphonates (known as Rp-Pm and Sp-Pm) single substitutions for each phosphate of d(GACpGpApApTpTpCpGTC) have decreased the binding affinity as well as the enzymatic cleavage [30]. R- or S-phosphorothioates (known as R-Ps or S-Ps) have produced significant stereospecific effects on *Eco*RI binding and cleavage [33] [37] [45]. The cleavage inhibition has been considered as the consequence of structural alteration such as a minor displacement of Asp91 and Glu111 [33]. Substitutions of *O*-ethyl phosphotriester derivative have also demonstrated a position-dependent resistance to enzymatic cleavage [45], although ethylation of this phosphate weakly interfered with binding of the enzyme [46]. The absolute configuration assigned as Rp has shown that the ethoxy group was oriented into the major groove of the DNA molecule (“inward” orientation), whereas the Sp configuration was oriented “outward” of the DNA molecule [45]. Structure analysis has shown that the phosphate group between two thymine residues was located almost directly across the scissile bond on the complementary strand, therefore, it was proposed the esterification of this phosphate group could influence the cleavage of the scissile bond on the opposite strand rather than on the modified strand. To prove it, Koziolkiewicz and Stec have used heteroduplexes of d(AAGAATTCCC)/d(GGGAATTCTT) and d(AAGAATp_(Oet)_TCCC)/d(GGGAATTCTT). Results have shown that the presence of an *O*-ethyl group between the two thymine residues in strand d(AAGAATp_(Oet)_TCCC) inhibited the cleavage of the opposite unmodified strand more effectively than cleavage of the modified strand [45].

As shown in this study, the 2′-OMeN substitution for T5 residue on the template strand has completely inhibited the cleavage. It was interesting because the inhibitoriest effect occurred at the T5 residue rather than the A2 residue (Fig. 5). The same phenomenon was also observed when the 2′-OMeN substitution for T5 residue on the F-ON strand (data not shown). The 2′-OMeN substitution for A2 residue on the F-ON strand reduced the catalytic activity significantly although not completely, and the double-substitution of 2′-OMeN for T5 residues on both strands exhibited a similar 100% inhibition (data not shown). By analogy to the Koziolkiewicz and Stec’s conclusion, we speculated that the T5 residue might transfer the sugar pucker conformational changes to the unmodified strand through the interstrand T5-A2 basepair interaction, inhibiting the cleavage of the unmodified strand. Further investigations are needed to prove this speculation.

### Biological Significance

Synthetic biology has become very attractive as an emerging field. Synthetic biology utilizes extensively tool enzymes, such as restriction endonucleases and ligases to construct new DNA sequences by cleaving and jointing nucleotide fragments. Therefore, assembling a biological circuit using different biological elements and parts is a challenging test, where repetitive cleavage and jointing will be performed. Thus, enhancement of cleavage efficiency under one condition and repression of cleavage under another condition could offer operation flexibility to optimize the new DNA constructs. Our study shows that substitutions of 2′-OMeN, 2′-FNA and LNA for nucleotides of *Eco*RI recognition sequence could provide the feasibility to regulate the cleavage efficiency of *Eco*RI in varied extent *in vitro*. It is our hope to exploit this kind of characteristics of C2′-modified nucleotide derivatives in synthetic biology.

## Conclusions

We have conducted a study on the effect of 2′-OMeN substituted substrates on the *Eco*RI enzymatic activity. Our results showed that 2′-OMeN substitutions for the *Eco*RI recognition sequence could decrease the cleavage efficiency for A2, A3 and T4 substitutions, and preserve the original cleavage efficiency for G1 and C6 substitutions. 2′-OMeN substitution for T5 residue, however, diminished the enzymatic activity completely. LNA and 2′-FNA also demonstrated similar results. The distorted phosphate backbone by a switch from C2′-endo to C3′-endo sugar pucker conformation might be responsible for these observed cleavage activity changes.
